# Association between progression-free survival and overall survival in women receiving first-line treatment for metastatic breast cancer: evidence from the ESME real-world database

**DOI:** 10.1186/s12916-023-02754-5

**Published:** 2023-03-08

**Authors:** Coralie Courtinard, Sophie Gourgou, William Jacot, Matthieu Carton, Olivier Guérin, Laure Vacher, Aurélie Bertaut, Marie-Cécile Le Deley, David Pérol, Patricia Marino, Christelle Levy, Lionel Uwer, Geneviève Perrocheau, Renaud Schiappa, Florence Bachelot, Damien Parent, Mathias Breton, Thierry Petit, Thomas Filleron, Agnès Loeb, Simone Mathoulin-Pélissier, Mathieu Robain, Suzette Delaloge, Carine Bellera

**Affiliations:** 1 University of Bordeaux, Inserm, Bordeaux Population Health Research Center, Epicene Team, UMR 1219, 33000 Bordeaux, France; 2 Unicancer, 101 Rue de Tolbiac, 75654 Paris, France; 3 Biometrics Unit, Institut du Cancer de Montpellier, 208 Rue Des Apothicaires, 34298 Montpellier, France; 4 University of Montpellier, 163 Rue Auguste Broussonnet, 34090 Montpellier, France; 5 Department of Medical Oncology, Institut du Cancer de Montpellier, 208 Rue Des Apothicaires, 34298 Montpellier, France; 6 Institut de Recherche en Cancérologie de Montpellier (IRCM) INSERM U1194, 208 Rue Des Apothicaires, 34298 Montpellier, France; 7 Department of Biostatistics, Institut Curie, 35 Rue Dailly, 92210 Saint-Cloud, France; 8 Department of Medical Information, Institut de Cancérologie de L’Ouest Nantes & Angers, 15 Rue André Boquel, 49055 Angers, France; 9 Department of Medical Oncology, Centre Jean Perrin, 58 Rue Montalembert, 63011 Clermont Ferrand, France; 10 Department of Biometry, Institut de Cancérologie de Bourgogne, 21079 Dijon, France; 11 Centre Oscar Lambret, 3 Rue Frédéric Combemale, 59000 Lille, France; 12 Department of Biometry, Centre Léon Bérard, 28 Prom. Léa Et Napoléon Bullukian, Lyon, 69008 France; 13 Institut Paoli-Calmettes, SESSTIM UMR912, 232, Boulevard Sainte-Marguerite, 13009 Marseille, France; 14 Aix-Marseille Université, Inserm, IRD, SESSTIM Sciences Économiques Et Sociales de La Santé Et Traitement de L’information Médicale, 13009 Marseille, France; 15 Department of Medical Oncology, Centre François Baclesse, 3 Avenue du Général Harris, 14000 Caen, France; 16 Medical Oncology Department, Institut de Cancérologie de Lorraine, 6 Avenue de Bourgogne, 54519 Vandœuvre-lès-Nancy, France; 17 Department of Pharmacy, Institut de Cancérologie de L’Ouest Nantes, Bd Professeur Jacques Monod, 44800 Saint-Herblain, France; 18 Department of Biometry, Centre Antoine Lacassagne, 33 Avenue de Valambrose, 06189 Nice, France; 19 Department of Medical Information, Institut Curie, 26 Rue d’Ulm, 75005 Paris, France; 20 Department of Pharmacy, Institut de Cancérologie Jean-Godinot, 1 Rue du Général Koenig, 51100 Reims, France; 21 Department of Medical Information, Centre Eugène Marquis, Avenue de La Bataille Flandres-Dunkerque, 35000 Rennes, France; 22 Department of Medical Oncology, Institut de Cancérologie Strasbourg Europe (ICANS), 17 Rue Albert Calmette, 67200 Strasbourg, France; 23 Department of Biometry, Institut Claudius Regaud – IUCT Oncopole, 1 Avenue Irène-Joliot-Curie, 31059 Toulouse, France; 24 Department of Medical Information, Centre Henri Becquerel, Rue d’Amiens, 76000 Rouen, France; 25 Inserm CIC1401, Clinical and Epidemiological Research Unit, Institut Bergonié, Comprehensive Cancer Center, 33000 Bordeaux, France; 26 Department of Cancer Medicine, Gustave Roussy Cancer Campus, 114 Rue Edouard Vaillant, 94800 Villejuif, France

**Keywords:** Real-word data, Breast cancer, Overall survival, Progression-free survival, Association, Surrogacy

## Abstract

**Background:**

Overall survival (OS) is the gold standard endpoint to assess treatment efficacy in cancer clinical trials. In metastatic breast cancer (mBC), progression-free survival (PFS) is commonly used as an intermediate endpoint. Evidence remains scarce regarding the degree of association between PFS and OS. Our study aimed to describe the individual-level association between real-world PFS (rwPFS) and OS according to first-line treatment in female patients with mBC managed in real-world setting for each BC subtype (defined by status for both hormone-receptor [HR] expression and HER2 protein expression/gene amplification).

**Methods:**

We extracted data from the ESME mBC database (NCT03275311) which gathers deidentified data from consecutive patients managed in 18 French Comprehensive Cancer Centers. Adult women diagnosed with mBC between 2008 and 2017 were included. Endpoints (PFS, OS) were described using the Kaplan–Meier method. Individual-level associations between rwPFS and OS were estimated using the Spearman’s correlation coefficient. Analyses were conducted by tumor subtype.

**Results:**

20,033 women were eligible. Median age was 60.0 years. Median follow-up duration was 62.3 months. Median rwPFS ranged from 6.0 months (95% CI 5.8–6.2) for HR-/HER2 − subtype to 13.3 months (36% CI 12.7–14.3) for HR + /HER2 + subtype. Correlation coefficients were highly variable across subtypes and first-line (L1) treatments. Among patients with HR − /HER2 − mBC, correlation coefficients ranged from 0.73 to 0.81, suggesting a strong rwPFS/OS association. For HR + /HER2 + mBC patients, the individual-level associations were weak to strong with coefficients ranging from 0.33 to 0.43 for monotherapy and from 0.67 to 0.78 for combined therapies.

**Conclusions:**

Our study provides comprehensive information on individual-level association between rwPFS and OS for L1 treatments in mBC women managed in real-life practice. Our results could be used as a basis for future research dedicated to surrogate endpoint candidates.

**Supplementary Information:**

The online version contains supplementary material available at 10.1186/s12916-023-02754-5.

## Background

Despite a decrease of the mortality rate, breast cancer (BC) is still the major cause of cancer death among women worldwide [1]. BC is a heterogeneous disease and can be classified according to the tumor immunohistochemical profile, characterized by the presence or absence of hormone-receptor expression (HR-positive [HR +]/HR-negative [HR −] status) and/or human epidermal growth factor receptor 2 (HER2) protein expression and/or HER2gene amplification (HER2 − positive [HER2 +]/HER2 − negative status [HER2 −]) in the tumor cells. Up to 30% of BC patients will experience distant metastases over their lifetime [2]. Metastatic breast cancer (mBC) is a disease with poor prognosis, with median overall survival (OS) ranging from 14.8 months for triple-negative mBC (TN mBC, defined as the lack of HR expression and of HER2 over-expression/amplification) to around 5 years for HER2 + disease respectively [3]. The selection of the most appropriate treatment depends on the characteristics of the patient (age, performance status), the adjuvant treatment (type and duration of therapies for early BC), and the metastatic disease (number of metastatic sites, type of involved organs, time to metastatic occurrence, molecular profile). In the metastatic setting, chemotherapy, endocrine therapy, or targeted therapy are the recommended treatments, as induction or maintenance treatment, according to the tumor molecular profile [4].

In cancer randomized controlled trials (RCT), drug efficacy is assessed using OS, considered as the gold standard efficacy endpoint [5–7]. However, in the advanced setting, the multiple lines of treatments may affect OS and thus bias the assessment of the true treatment effect. In addition, observing a benefit on OS may require a large number of patients and extensive follow-up, limiting the feasibility of clinical trials based on OS as the primary outcome. In this context, alternative endpoints that could capture treatment benefit accurately and be measurable earlier is central for the evolution of clinical research in oncology. Progression-free survival (PFS), although recognized as presenting some limitations [8], has been used increasingly over the past decades [9] and is now the most common primary efficacy endpoint in mBC clinical trials [10]. The use of PFS relies on the hypothesis that it can adequately replace OS, i.e., be a valid surrogate of OS; otherwise, this might lead to the marketing of drugs that do not ultimately improve OS [11, 12]. PFS however has not been validated as a surrogate endpoint in the context of mBC [13].

Real-world data (RWD) are defined as observational data from other sources than clinical trials, such as electronic medical records, registries, insurance claims, pharmacy records, death certificates, and other patient-generated data [14]. RWD bring information on patient’s profiles not included in RCT and supplement with real-life knowledge on patient management, treatment strategies, and long-term survival. As such, they complement results of RCT by allowing one (i) to assess the generalizability of survival outcomes reported in RCT to the real-life setting, (ii) to expand generalizability of trials’ results to underrepresented or specific populations, and (iii) to generate scientific hypotheses. In mBC, the availability of large datasets for researchers such as the longitudinal Epidemiology Strategy and Medical Economics (ESME) mBC Database are a unique opportunity to investigate real-life survival outcomes for distinct subgroups of mBC patients. These could subsequently be used to validate efficacy data observed in published RCT or generate study hypotheses when estimating sample size for a future RCT. ESME-mBC-derived data have been published, either to describe treatment patterns and patient outcomes for some specific subgroups [15–20] or to report specifically on OS and associated prognostic factors in mBC [3, 21, 22].

Our primary objective was to describe the individual-level association between rwPFS and OS according to first-line (L1) treatment in women treated for mBC as a potential surrogate endpoint. Secondary objectives included description of treatment patterns, rwPFS and OS, overall and by mBC subtype.

## Methods

### Data source

The Epidemio-Strategy and Medical Economic (ESME) Research Program is a French academic initiative supporting the centralization of structured and non- structured data documented in the electronic health records (EHR) (clinical notes, pathology reports and radiology reports) of patients treated for malignant conditions in a unique secured web-based data platform available for researchers. The ESME mBC data platform is an EHR-derived database that gathers exhaustive data on consecutive patients who initiated a L1 treatment for mBC between 01 January 2008 and 31 December 2017 in one of 18 French Comprehensive Cancer Centers (*clinicaltrials.gov; NCT 03,275,311*). Patients who only received surgery of a breast-related metastatic lesion were not eligible for selection into the ESME mBC database. The full methodology is described elsewhere [23]. Data extraction took place on April 14, 2020, and the extracted dataset included deidentified individual data for about 23,000 patients, with up to a 12-year follow-up. Available data were demographics, tumor characteristics, clinical features, clinical events, and treatments.

### Study population

We included all female patients older than 18 years diagnosed with mBC (de novo disease or first metastatic recurrence) between January 1, 2008, and December 31, 2017, and who received a L1 systemic treatment such as chemotherapy, endocrine therapy or targeted therapy, whatever the sequence (monotherapy or combination of therapies using distinct mechanisms of actions, i.e., polytherapy). A treatment line was defined as all anti-cancer treatments received in the absence of tumor progression. We excluded patients without informative data for tumor subtype (e.g., status for both HR expression and HER2 expression/gene amplification). Patients receiving radiation therapy or anti-resorptive drugs (e.g., bisphosphonates, denosumab) as unique treatment were not considered in the analysis. Patients were excluded if a second breast cancer was diagnosed before the onset of metastatic disease in order to limit potential inconsistencies between both breast cancer tumor subtypes and the metastases.

### Variables and definitions

Age, tumor characteristics (histological grade, histologic type, HR status, HER2 status), the dates of first disease progression, start of metastatic lines of treatment, and last contact were derived from clinical patient records using standard definitions validated by the ESME Scientific Group.

HER2 status and HR status were derived from existing results about metastatic tissue sampling where available, or, if not available, from last sampling on early disease. Tumors were defined as HR positive (HR +) if estrogen receptor (ER) or progesterone receptor (PR) expression was >  = 10% (immunohistochemistry), as per European guidelines. HER2 immunohistochemical (IHC) score 3 + or IHC score 2 + with a positive fluorescence in situ hybridization (FISH) or chromogenic in situ hybridization (CISH) classified the cancer as HER2 positive (HER2 +). On the other hand, all cancers with an IHC score 0–1 + or 2 + with a negative FISH/CISH test, as well as patients with a negative FISH/CISH test without IHC information, were considered as HER2 negative (HER2 −). Cancers with an IHC score 2 + without FISH/CISH test information were considered as HER2 indeterminate.

The four tumor subtypes are described as follows: triple-negative breast cancer was defined as ER expression < 10% and PR expression < 10% and HER2 non overexpressed and/or non amplified (TN mBC). Hormone receptor-positive and HER2-positive BC was defined as ER and/or PR >  = 10% and HER2 protein overexpression (3 +) and/or gene amplification (HR + /HER2 + mBC). Hormone receptor-negative and HER2-positive BC was defined by ER and PR expression < 10% and HER2 protein overexpression (3 +) and/or gene amplification (HR − /HER2 + mBC). Finally, hormone receptor-positive (HR +) and HER2-negative (HER2 −) BC was defined by an expression of ER and/or PR >  = 10% and no overexpression nor amplification of HER2 (HR + /HER2 − mBC).

De novo metastatic disease was considered when the first occurrence of metastatic disease was diagnosed within 6 months after the diagnosis of the primary BC. Metastasis-free interval (MFI) was defined as the time between initial diagnosis and metastatic relapse.

Histological grade and histologic type were derived using the first informative results on the primary tumor whatever breast surgical procedures (biopsy, tumorectomy, lumpectomy, and mastectomy).

The number of metastatic sites was calculated based on the number of organs involved with one or more metastases diagnosed within 1 month (30 days) from the diagnosis of the first metastatic occurrence.

The first-line (L1) therapy was defined as intravenous or per os therapeutic regimen (chemotherapy, targeted therapy, immunotherapy, and/or endocrine therapy) initiated at the metastatic diagnosis or within 12 weeks following it. Drug classifications are listed in Additional file 1 Table S1.

L1 treatment patterns were defined according to the therapeutic classes of drugs: chemotherapy only, targeted therapy only, endocrine therapy only, chemotherapy and endocrine therapy, chemotherapy and targeted therapy, chemotherapy, targeted therapy and endocrine therapy, endocrine therapy and targeted therapy, immunotherapy-based regimen, and other therapy. Anthracyclines, purine analogs, pyrimidine analogs, alkylating agents, platinum derivatives, taxanes, and vinca alkaloids were classified as “chemotherapy.” Protein kinase inhibitors, vascular endothelial growth factor (VEGF)/vascular endothelial growth factor receptor inhibitors, and other agents were classified as “targeted therapy.” Endocrine therapy was assigned for aromatase inhibitors, anti-estrogens, and luteinizing hormone-releasing hormone agonists.

For non-de novo mBC patients, the type of adjuvant therapy received at early BC stage was also described.

The first disease progression was derived using diagnosed clinical events recorded in the database (local relapse, progression in the involved organs, metastases in a new organ) and progression-related reason(s) for termination of drugs included in the L1 therapy.

rwPFS was defined as the time from initial diagnosis of mBC to the date of disease progression (regional recurrence, progression, appearance/occurrence of metastases and distant recurrence) or death (any cause), whichever came first. OS was defined as the time from diagnosis of mBC to the date of death from any cause.

The present study was validated by the ESME mBC Scientific Group. No formal dedicated informed consent was required but all patients had been informed about the re-use of their electronically recorded data in compliance with the General Data Protection *Regulation*. The ESME mBC database was authorized by the French data protection authority (Registration ID 1,704,113; authorization N°DE-2013.-117; complementary authorization was obtained on 14 October 2019 regarding the ESME research Data Warehouse). The analysis was approved by an independent ethics committee (Comité De Protection Des Personnes Sud-Est II- 2015–79).

### Statistical analyses

Baseline characteristics were summarized using frequency and percentage for qualitative variables. Median and inter-quartile range were reported for quantitative variables. We reported frequencies and proportions for variables with missing or not documented information. No statistical test was performed for the descriptive analyses.

Median follow-up was estimated using the reverse Kaplan–Meier Method [24]. Survival data were estimated using Kaplan–Meier method and we reported median survival times with their respective 95% confidence interval (95%CI). Data for patients without the events of interest were censored at the date of last contact recorded in the database.

We estimated the individual-level association between rwPFS and OS using a Spearman rank correlation coefficient expressed as a value between 0 (no association) and 1 (perfect association) with 95% CI. Copula models allow one to jointly model two time-to-event variables [25]. We used a reviewed copula-based approach that introduced an iterative multiple imputation method for the estimation of the correlation coefficient [26]. The strength of the rwPFS/OS association was ranged according to the estimated correlation coefficient as follows: 0–0.19 was considered as very weak, 0.2–0.39 as weak, 0.40–0.59 as moderate, 0.6–0.79 as strong, and 0.8–1 as very strong correlation [27]. We estimated and reported individual rwPFS/OS associations according to mBC subtype and first-line mBC treatment.

Data were analyzed using R software (v 3.6.1).

## Results

### Characteristics and treatments

**Fig. 1:**
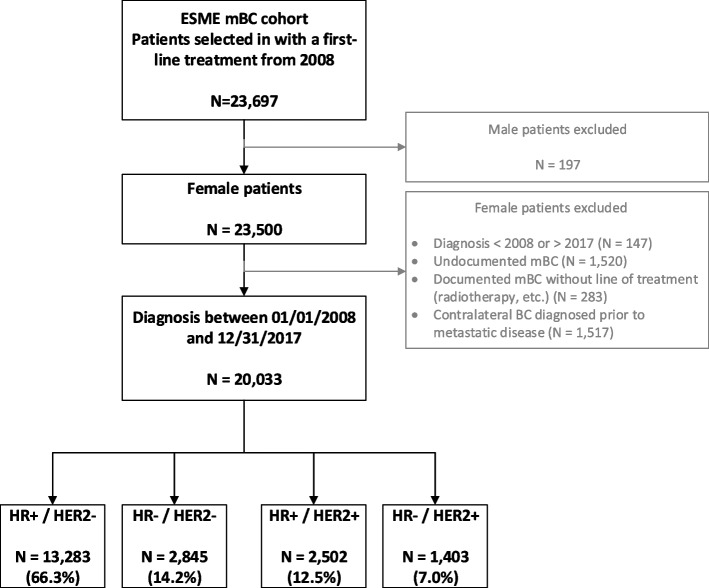
Study flow chart ESME, epidemiological strategy and medical economics; mBC, metastatic breast cancer; HR + , presence of hormone receptor; HR − , absence of hormone receptor; HER2 + , human epidermal growth factor receptor 2 (HER2) protein overexpression; HER2 − , no HER2 protein overexpression. TN, triple negative

On the date of data extraction, the ESME mBC database included a total of 23,697 mBC subjects. Of those, 20,033 satisfied the eligibility criteria for our study population (Fig. 1). A total of 18 239 patients (91.0%) had at least one IHC score assessment for the primary tumor, and 1852 (9.2%) had at least one IHC score assessment for metastasis. The distribution of mBC subtypes was as follows: 66.3% for HR + /HER2 − (13 283 patients), 14.2% for TN (2 845 patients), 12.5% for HR + /HER2 + (2 502 patients), and 7.0% (1 403 patients) for HR − /HER2 + .

Clinical and tumor characteristics are presented overall and by tumor subtype (Table 1). De novo mBC was highly represented among HR − /HER2 + mBC patients (49.0%) and HR + /HER2 + mBC patients (40.6%). Most patients had two metastatic sites (79.0%). Overall, bone metastases were the most frequent (57.9%, ranging from 35.4% [TN mBC] to 65.3% [HR + /HER2 − mBC]), with HR − /HER2 + mBC patients presenting most often with liver metastases (38.9%) and TN mBC patients with metastatic lymph nodes (43.4%).

**Table 1:** Patient characteristics according to tumor subtype

	**HR + /HER2 − ** **(*N* = 13,283)**	**TN** **(*N* = 2845)**	**HR + /HER2 + ** **(*N* = 2502)**	**HR − /HER2 + ** **(*N* = 1403)**	**Total** **(*N* = 20,033)**
Age (years) (median, [IQR])	62.0 (51.0, 71.0)	56.0 (45.0, 66.0)	57.0 (47.0, 67.0)	56.0 (46.5, 64.0)	60.0 (50.0, 70.0)
Personal history of other cancer	401 (3.0%)	111 (3.9%)	75 (3.0%)	38 (2.7%)	625 (3.1%)
Histologic type (primary tumor)					
IDC	9498 (71.5%)	2382 (83.7%)	2045 (81.7%)	1185 (84.5%)	15,110 (75.4%)
ILC	2263 (17.0%)	123 (4.3%)	168 (6.7%)	44 (3.1%)	2598 (13.0%)
IDC + ILC	149 (1.1%)	8 (0.3%)	28 (1.1%)	4 (0.3%)	189 (0.9%)
Other types	1173 (8.8%)	296 (10.4%)	227 (9.1%)	155 (11.0%)	1851 (9.2%)
Not documented	200 (1.5%)	36 (1.3%)	34 (1.4%)	15 (1.1%)	285 (1.4%)
Histological grade (primary tumor)					
Grade 1	1532 (11.5%)	46 (1.6%)	132 (5.3%)	16 (1.1%)	1726 (8.6%)
Grade 2	6883 (51.8%)	758 (26.6%)	1125 (45.0%)	459 (32.7%)	9225 (46.0%)
Grade 3	2775 (20.9%)	1662 (58.4%)	866 (34.6%)	724 (51.6%)	6027 (30.1%)
Not documented	2093 (15.8%)	379 (13.3%)	379 (15.1%)	204 (14.5%)	3055 (15.2%)
De novo mBC disease					
Yes	4217 (31.7%)	872 (30.7%)	1017 (40.6%)	687 (49.0%)	6793 (33.9%)
No	9066 (68.3%)	1973 (69.3%)	1485 (59.4%)	716 (51.0%)	13,240 (66.1%)
*For patients without *de novo* mBC disease: metastasis free interval [MFI]*					
− [6:24[	1109 (8.3%)	1043 (36.7%)	235 (9.4%)	280 (20.0%)	2667 (13.3%)
− [24:48[	1939 (14.6%)	484 (17.0%)	449 (17.9%)	238 (17.0%)	3110 (15.5%)
− [48:96[	2528 (19.0%)	224 (7.9%)	423 (16.9%)	121 (8.6%)	3296 (16.5%)
− [96:120[	955 (7.2%)	42 (1.5%)	112 (4.5%)	29 (2.1%)	1138 (5.7%)
− > = 120	2535 (19.1%)	180 (6.3%)	266 (10.6%)	48 (3.4%)	3029 (15.1%)
*For patients without *de novo mBC disease, therapy used in adjuvant setting (*)					
IV chemotherapy	6094 (67.2%)	1752 (88.8%)	1136 (76.5%)	601 (83.9%)	9583 (72.4%)
Oral chemotherapy	149 (1.6%)	80 (4.1%)	52 (3.5%)	27 (3.8%)	308 (2.3%)
Endocrine therapy	7582 (83.6%)	76 (3.9%)	1175 (79.1%)	30 (4.2%)	8863 (66.9%)
Number of metastatic sites at mBC diagnosis					
< 3	10,627 (80.0%)	2135 (75.0%)	1984 (79.3%)	1077 (76.8%)	15,823 (79.0%)
> = 3	2656 (20.0%)	710 (25.0%)	518 (20.7%)	326 (23.2%)	4210 (21.0%)
Brain/CNS/CSF metastases	543 (4.1%)	359 (12.6%)	227 (9.1%)	201 (14.3%)	1330 (6.6%)
Bone metastases	8678 (65.3%)	1007 (35.4%)	1375 (55.0%)	541 (38.6%)	11,601 (57.9%)
Liver metastases	3378 (25.4%)	787 (27.7%)	802 (32.1%)	546 (38.9%)	5513 (27.5%)
Lung metastases	2783 (21.0%)	1024 (36.0%)	639 (25.5%)	420 (29.9%)	4866 (24.3%)
Metastatic lymph nodes	3458 (26.0%)	1235 (43.4%)	751 (30.0%)	506 (36.1%)	5950 (29.7%)
Pleural metastases	1444 (10.9%)	296 (10.4%)	178 (7.1%)	108 (7.7%)	2026 (10.1%)

(*) Percentages may not sum up to 100% as patients may receive more than one adjuvant treatment

*Abbreviations*: *IQR*, interquartile range; *BC*, breast cancer; *mBC*, metastatic breast cancer; *HR* + , presence of hormone receptor; *HR − *, absence of hormone receptor; *HER2* + , human epidermal growth factor receptor 2 (HER2) protein overexpression; *HER2 − *, no HER2 protein overexpression; *TN*, triple negative; *IDC*, invasive ductal carcinoma; *ILC*, invasive lobular carcinoma; *IV*, intravenous; *PO*, per os; *CNS*, central nervous system; *CSF*, cerebrospinal fluid

First-line treatments are described in Table 2. First-line treatments with a focus on anti-HER2 therapies are reported in Additional file 1 Table S2. Number of lines of treatment by tumor subtype are reported in Additional file 1 Table S3.

**Table 2:** Therapeutic strategy during first-line therapy for mBC disease

	**HR + /HER2 − (*N* = 13,283)**	**TN** **(*N* = 2845)**	**HR + /HER2 + (*N* = 2502)**	**HR − /HER2 + ** **(*N* = 1403)**	**Total (*N* = 20,033)**
Chemotherapy only	1631 (12.3%)	1804 (63.4%)	84 (3.4%)	75 (5.3%)	3594 (17.9%)
Targeted therapy only	8 (0.1%)	6 (0.2%)	53 (2.1%)	116 (8.3%)	183 (0.9%)
Endocrine therapy only	5545 (41.7%)	39 (1.4%)	342 (13.7%)	7 (0.5%)	5933 (29.6%)
Chemotherapy and endocrine therapy	3383 (25.5%)	46 (1.6%)	85 (3.4%)	3 (0.2%)	3517 (17.6%)
Chemotherapy and targeted therapy	699 (5.3%)	921 (32.4%)	674 (26.9%)	1164 (83.0%)	3458 (17.3%)
Chemotherapy, targeted therapy and endocrine therapy	1518 (11.4%)	15 (0.5%)	1036 (41.4%)	33 (2.4%)	2602 (13.0%)
Endocrine therapy and targeted therapy	492 (3.7%)	1 (0.0%)	228 (9.1%)	5 (0.4%)	726 (3.6%)
Immunotherapy-based regimen	6 (0.0%)	13 (0.5%)	0 (0.0%)	0 (0.0%)	19 (0.1%)
Other therapy	1 (0.0%)	0 (0.0%)	0 (0.0%)	0 (0.0%)	1 (0.0%)

*Abbreviations*: *mBC*, metastatic breast cancer; *HR* + , presence of hormone receptor; *HR − *, absence of hormone receptor; *HER2* + , human epidermal growth factor receptor 2 (HER2) protein overexpression; *HER2 − *, no HER2 protein overexpression; *TN*, triple negative

### Outcomes

**Fig. 2:**
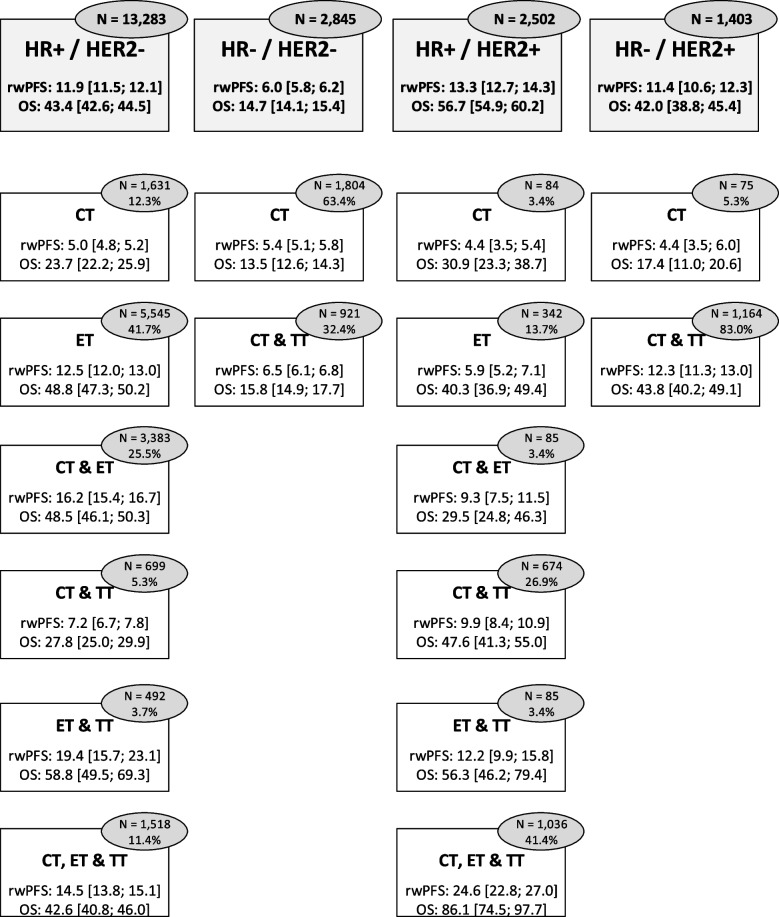
Median survival outcomes (overall survival [OS], real-world progression-free-survival [rwPFS]) in metastatic breast cancer (mBC) patients after diagnosis of mBC, according to first-line treatment by tumor subtype (in months) HR + , presence of hormone receptor; HR − , absence of hormone receptor; HER2 + , human epidermal growth factor receptor 2 (HER2) protein overexpression; HER2 − , no HER2 protein overexpression. CT, chemotherapy only; ET, endocrine therapy only; TT, targeted therapy only; CT& ET, chemotherapy and endocrine therapy; CT and TT, chemotherapy and targeted therapy; ET & TT, endocrine therapy and targeted therapy; CT, TT and ET, chemotherapy, targeted therapy and endocrine therapy. Median estimates are presented in months, with 95% confidence interval (95% CI)

The median follow-up duration was 62.3 months (95% CI 58.4–63.6). Median time from initial diagnosis of mBC to the initiation of first-line treatment was 19 days, ranging from 18 days for HR + mBC to 25 days for TNBC. Survival outcomes are summarized in Fig. 2, by tumor subtype and first-line treatment.

**Fig. 3:**
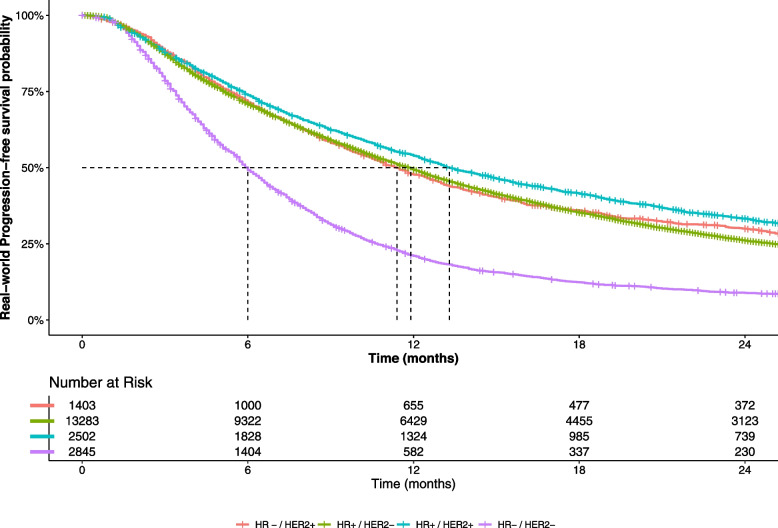
Estimated real-world progression-free survival curve, by tumor subtype, for study population (*N* = 20,033) HR + , presence of hormone receptor; HR − , absence of hormone receptor; HER2 + , human epidermal growth factor receptor 2 (HER2) protein overexpression; HER2 − , no HER2 protein overexpression; TN, triple negative

Median rwPFS under first line treatment was 10.6 months (95% CI 10.4–10.8) for the whole population: 6.0 months (95% CI 5.8–6.2) for TN mBC patients, 11.4 months (95% CI 10.6–12.3) for HR − /HER2 + mBC patients, 11.9 months (95% CI 11.5–12.1) for HR + /HER2 − mBC patients, and 13.3 months (95% CI 12.7–14.3) for HR + /HER2 + mBC patients. rwPFS curves are reported in Fig. 3.

**Fig. 4:**
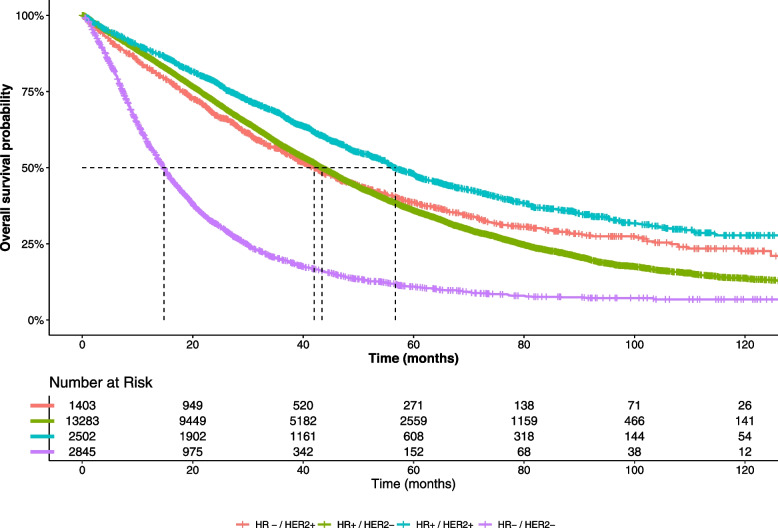
Estimated overall survival curve, by tumor subtype for study population (*N* = 20,033) OS, overall survival; HR + , presence of hormone receptor; HR − , absence of hormone receptor; HER2 + , human epidermal growth factor receptor 2 (HER2) protein overexpression; HER2 − , no HER2 protein overexpression; TN, triple negative

Median OS was 39.5 months (95% CI 38.7–40.5) for the whole population: 14.7 months (95% CI 14.1–15.4) for TN mBC patients, 42.0 months (95% CI 38.8–45.4) for HR − /HER2 + mBC patients, 43.4 (95% CI 42.6–44.5) for HR + /HER2 − mBC patients, and 56.7 months (95% CI 54.9–60.2) for HR + /HER2 + mBC patients. OS curves are shown in Fig. 4.

### rwPFS-OS correlations

Individual-level associations between rwPFS and OS are presented in Table 3. Associations ranged from very weak to very strong, with correlation coefficients ranging from 0.33 (95% CI 0.12–0.52) for HR + /HER2 + mBC women treated with chemotherapy to 0.81 (95% CI 0.79–0.82) for TN mBC women treated with chemotherapy. High variability was observed for HR-positive subgroups with weak to strong associations. For HR + /HER2 + mBC patients, the correlation coefficient ranged from 0.33 to 0.43 when a single type of therapy was used (chemotherapy or endocrine), while it was 0.67 or more when multiple types of therapy were combined. For both TN and HR − /HER2 + subgroups, associations were at least strong. Among the 1804 patients with TN mBC treated with chemotherapy, the estimate was 0.81 (95% CI, 0.79–0.82). When targeted therapy was added to the initial chemotherapy regimen, the estimated correlation was 0.73.

**Table 3:** Individual-level association between real-world progression-free survival and overall survival in metastatic breast cancer (mBC) patients after diagnosis of mBC, according to mBC subtype

	Patients	*ρ* coef	95% CI
*TN*			
Chemotherapy only	1804	0.81	0.79 − 0.82
Chemotherapy and targeted therapy	921	0.73	0.69 − 0.76
*HR* + */HER2* +			
Chemotherapy only	84	0.33	0.12 − 0.52
Endocrine therapy only	342	0.43	0.32 − 0.53
Chemotherapy and endocrine therapy	85	0.72	0.58 − 0.82
Chemotherapy and targeted therapy	674	0.67	0.61 − 0.72
Chemotherapy, targeted therapy, and endocrine therapy	1036	0.78	0.74 − 0.82
Endocrine therapy and targeted therapy	228	0.71	0.61 − 0.78
*HR − /HER2* +			
Chemotherapy only	75	0.67	0.51 − 0.78
Chemotherapy and targeted therapy	1164	0.81	0.78 − 0.84
*HR* + */HER2 − *			
Chemotherapy only	1631	0.58	0.54 − 0.61
Endocrine therapy only	5545	0.66	0.64 − 0.68
Chemotherapy and endocrine therapy	3383	0.78	0.76 − 0.79
Chemotherapy and targeted therapy	699	0.45	0.39 − 0.51
Chemotherapy, targeted therapy and endocrine therapy	1518	0.70	0.67 − 0.73
Endocrine therapy and targeted therapy	492	0.61	0.51 − 0.70

*Abbreviations*: *mBC*, metastatic breast cancer; *HR* + , presence of hormone receptor; *HR − *, absence of hormone receptor; *HER2* + , human epidermal growth factor receptor 2 (HER2) protein overexpression; HER2 − , no HER2 protein overexpression; *TN*, triple negative

Correlations are expressed as Spearman’s *ρ* coefficient with 95% confidence interval (95% CI)

## Discussion

This retrospective analysis provided estimates on individual-level associations between rwPFS and OS in 20,033 women diagnosed in 2008–2017 with mBC who initiated first-line anti-cancer treatment. This large national cohort was a unique opportunity to report on individual-level associations between rwPFS and OS for each L1 treatment pattern, overall and by tumor subtype. Individual-level associations between rwPFS and OS were at least strong for both TN and HR − /HER2 + subtypes (rank correlation coefficient equal or higher than 0.67). For HR + subgroups with considerable variety in therapeutic options, associations were highly variable (weak to strong) depending on the treatment. Overall, within the 4 mBC subgroups, individual-level correlation between rwPFS and OS were at least strong for each dominant treatment class (i.e., chemotherapy for TN mBC, endocrine therapy for HR + /HER2 − , and anti-HER2 in either HER2 + subgroups).

Formal validation of a surrogate endpoint relies on the assessment of both individual- and trial-level associations, the latter being available through meta-analyses of RCTs. As of today however, few surrogate endpoints have been identified [13] and assessment of PFS as a surrogate for OS in mBC is still relevant.

To our knowledge, we report results of the first study assessing individual-patient level association between rwPFS and OS in the largest observational cohort including consecutive patients treated for mBC in the real-world setting. We assessed individual correlation based on the rank correlation approach, a rigorous tool used in the reference method for endpoint surrogacy assessment [28, 29]. Although RWD are not the primary source used for assessing surrogacy, our data source, ESME mBC database, offered a large dataset of individual-patient data [IPD] homogenously centralized to assess multiple endpoints. Secondly, exploring the value of RWD in the search of candidate endpoint for OS surrogacy, our IPD were sourced from a significant quantity of high-quality data. The ESME mBC cohort represented 23,000 + adult patients (without any upper limit regarding the age) consecutively treated in the French 18 participating centers over a 12-year time period. The BC subtypes distribution and de novo mBC disease proportion were consistent with the other observational studies published [30–33]. We relied on this highly reliable dataset updated on an annual basis (including update on patient status maintained update to date in each center supported by the local use of National registry of death certificates), and this leads to an objective estimation of OS and a strong external validity. Well-designed databases may lead to valid information even if conventional RCTs are still the reference for evidence [34]. Our retrospective analysis considered all 1L therapy and estimated rwPFS are consistent with published data [35, 36]. Finally, over the few past years, the ESME mBC database was extensively used to support post-marketing requirements for heath technology assessment body in France and to supplement clinical data package for marketing authorization file in Europe [37–42].

The comparison of our findings with published data on surrogate endpoints is complex due to differences in terms of statistical methods (meta-analytical approaches of RCTs using either IPD or aggregated data), recruitment period, distinct target populations (defined by HR expression or HER2 status), or distinct settings (first-line or subsequent lines of therapy). In the study published by Sherrill and al., individual-level association was weak (*ρ* = 0.38) based on 67 RCTs designed to assess different mBC therapies (mainly chemotherapy or endocrine therapy in monotherapy or combination) [43]. In a meta-analysis of RCTs assessing different types of therapy (including chemotherapy or endocrine therapy in monotherapy or combination) in multiple mBC setting and published between 1990 and 2020, moderate individual-level association was reported [44]. Based on the year of publication, most of RCTs (69.5% published earlier than 2005) were undertaken before our period of interest for mBC diagnosis (2008–2017) and 86.1% of RCTs included in the meta-analysis were not discriminant regarding the HR and HER2 status. Petrelli and al. reported a strong individual-level association (*ρ* = 0.81) in a meta-analysis of 20 RCTs assessing first-line targeted therapies in patients enrolled again before our period of interest [45]. All aforementioned meta-analyses were based on aggregated data and both PFS and time to progression merged as unique outcome when assessing correlation with OS at individual-patient level. Using IPD for meta-analyses, two studies reported individual-level association data between PFS and OS with a distinct definition for PFS [46, 47]. Burzykowski and al. reported a strong association (*ρ* = 0.81) at individual level in a meta-analysis of 11 RCTs comparing first-line anthracycline-based therapy with taxanes [46]. Again, no discrimination regarding the HR and HER2 status was retrieved among RCTs in the meta-analysis, which limits the comparison with our estimated associations for patients receiving chemotherapy alone with correlation coefficient ranging from 0.33 to 0.81 for HR + /HER2 + subtype and TN subtype respectively. The second meta-analysis investigated PFS surrogacy with OS in a set of 9 phase II/III RCTs evaluating anti-HER2 targeted therapies (trastuzumab or lapatinib), authors reported a strong individual-level association (*ρ* = 0.69) but 2 in 9 trials investigated drugs in second-line or more setting [47]. Our estimates of individual-level association between rwPFS and OS in HER2 + mBC patients exposed to L1 targeted therapy (range: 0.67–0.87) were consistent with those results focusing on drugs with similar mechanisms of action although all RCTs pooled in the meta-analysis included patients enrolled before 2008. As surrogacy assessment is specific to a disease and to the mechanisms of action of the drug, comparison of our results to published meta-analyses is complex as few of those are available. Investigations are supported using meta-analysis requiring large amount of individual patient data among terminated RCT assessing drug efficacy with the same mechanism of action.

Results of individual-patient level association using de-identified IPD collected in real-world setting are in line with published results seeking for PFS surrogacy with OS using RCTs data. This work shows the value of RWD to support the search of potential candidate surrogate endpoint for OS.

As RWD, the ESME mBC presents limitations [23, 48]. These include the lack of availability of electronic medical records data required to describe the global mBC management due to the low level of standardization of current electronic medical records as well as the retrospective patient selection-data collection. The interpretation of reported survival estimates may be limited by the presence of confounding factors inherent to the observational nature of RWD, as opposed to randomized controlled studies. As our primary objective was the association between rwPFS and OS, we only reported descriptive data for rwPFS and OS (crude estimates based on Kaplan–Meier) and refer the reader to earlier ESME mBC publications for further data on prognostic factors for rwPFS/OS (3, 21, 22). Concerning overall generalizability and external validity, the cohort centralizes data from patients treated in specialized cancer centers only, which thus may use different clinical practices compared with public hospitals and private institutions. Although we did assess real-world outcomes according to the first-line treatment by tumor subtypes, residual heterogeneity within treatment group remains. Indeed, we did not make any distinctions regarding the mechanism of action of the drugs among each treatment group, which affect individual-level association. As an example, we assigned to “targeted therapy” to both bevacizumab, VEGF-targeted therapy (component of mBC therapy affecting the tumor angiogenesis) and palbociclib, inhibitor of cyclin-dependent kinases 4 and 6 (CDK4/6) involved are the downstream of signaling pathways which lead to cellular proliferation). Antiangiogenics such as bevacizumab are unique in that PFS advantages appear to frequently be completely erased at OS, and this is the only therapy in breast cancer preclinically and clinically implicated to have a reversal effect in second and subsequent lines of therapy. This issue highlights that even within drug classes, residual variability in terms of mechanism of action may persist. Similarly, the impact of subsequent lines of treatment could be further investigated. Finally, in real-world setting, non-systematic RECIST-based progression evaluation may also introduce bias in our analyses, as compared to standardized RECIST-based assessment in RCTs.

## Conclusions

This study reports comprehensive information related to individual-level association between rwPFS and OS according to BC subtype and L1 mBC treatment. Overall, within the 4 mBC subgroups, individual-level correlation between rwPFS and OS were at least strong for each dominant treatment class (i.e., chemotherapy for TN mBC, endocrine therapy for HR + /HER2 − , and anti-HER2 in either HER2 + subgroups). Those results support the value of RWD when searching for candidate surrogate endpoints for OS. Data could be used subsequently to investigate or generate research hypotheses for future surrogate endpoint candidates. We also provided researchers with treatment patterns and rwPFS according to the 1L treatment received by tumor subtype. These estimates could be used when designing future research, in particular to provide information not readily available from the published trial literature for underrepresented populations.

### Supplementary Information

**Additional file 1:** **Table S1.** Drugclassification. **TableS2.** Therapeutic strategy during first-line therapyfor mBC disease for HER2+ mBC. **Table S3.** Number of metastatic lines of treatmentaccording to mBC subtype.

## Data Availability

The data that support the findings of this study are available from UNICANCER but restrictions apply to the availability of these data, which were used under license for the current study, and so are not publicly available. Data are however available from the authors upon reasonable request and with permission of UNICANCER.

## Abbreviations

BC: Breast cancer
CISH: Chromogenic in situ hybridization
HER: Electronic health records
ESME: Epidemiology Strategy and Medical Economics
FISH: Fluorescence in situ hybridization
HER2: Human epidermal growth factor receptor 2
HR: Hormone-receptor
IHC: Immunohistochemistry
IPD: Individual-patient data
L1: First-line
mBC: Metastatic breast cancer
PFS: Progression-free survival
OS: Overall survival
RW: Real-world
RCT: Randomized controlled trials
RWD: Real-world data
TN: Triple-negative
VEGF: Vascular endothelial growth factor
