# Supplementary material for: Association between progression-free survival and overall survival in women receiving first-line treatment for metastatic breast cancer: evidence from the ESME real-world database
Source: BMC Med. 2023 Mar 8;21:87. doi: 10.1186/s12916-023-02754-5 (PMC9993797; doi:10.1186/s12916-023-02754-5)
Supplement: Supplementary file 1 — Additional file 1: Table S1. Drugclassification. TableS2. Therapeutic strategy during first-line therapyfor mBC disease for HER2+ mBC. Table S3. Number of metastatic lines of treatmentaccording to mBC subtype. [file 12916_2023_2754_MOESM1_ESM.docx]

Additional file 1 :Supplementary tables S1-S3

Table S1: Drug classification

| Endocrine therapy | Abiraterone, anastrazole, exemestane, fulvestrant, goserelin, letrozole, leuprorelin, progesterone, megestrol, tamoxifen, toremifene et triptorelin. |
| --- | --- |
| Chemotherapy | azacitidine, bleomycin, cabazitaxel, capecitabine, carboplatin, cisplatin, cyclophosphamide, cytarabine, docetaxel, doxorubicin, epirubicin, eribulin, estramustine, etoposide, fluorouracil, gemcitabine, idrarubicin, ifosfamide, irinotecan, ixabepilone, melphalan, miltefosine, mitomycin, oxaliplatine, paclitaxel, temolozomide, thiotepa, topotecan, trabectedin, vinblastine, vincristine, vindesine, vinflunine, vinorelbine. |
| Targeted therapy | Abemaciclib, alpelisib, afatinib, aflibercept, bevacizumab, bortezomib, cetuximab, dasatinib, erlotinib, everolimus, gefitinib, lapatininb, masitinib, neratinib, nintedanib, niraparib, olaparib, palbociclib, panitumumab, panobinostat, pazopanib, pertuzumab, ribociclib, ridaforolimus, rucaparib, sorafenib, sunitinib, talazoparib, trastuzumab, trastuzumab-emtansine, vandetanib |
| Immunotherapy | atezolizumab, durvalumab and interleukins |

Table S2: Therapeutic strategy during first-line therapy for mBC disease for HER2+ mBC

|  | **HR+ / HER2+  (N=2502)** | **HR - / HER2+ (N=1403)** |
| --- | --- | --- |
| Chemotherapy only | 84 (3.4%) | 75 (5.3%) |
| Targeted therapy only | 53 (2.1%) | 116 (8.3%) |
| Anti-HER2 only | 52 (98.1%) | 115 (99.1%) |
| Targeted therapy than anti-HER-2 only | 1 (1.9%) | 1 (0.9%) |
| Endocrine therapy only | 342 (13.7%) | 7 (0.5%) |
| Chemotherapy & endocrine therapy | 85 (3.4%) | 3 (0.2%) |
| Chemotherapy & targeted therapy | 674 (26.9%) | 1164 (83.0%) |
| Chemotherapy & Anti-HER2 | 653 (96.9 %) | 1135 (97.5 %) |
| Chemotherapy & targeted therapy other than anti-HER-2 | 21 (3.1%) | 29 (2.5%) |
| Chemotherapy, targeted therapy & endocrine therapy | 1036 (41.4%) | 33 (2.4%) |
| Chemotherapy, Anti-HER2 & endocrine therapy | 996 (96.1%) | 32 (97.0%) |
| Chemotherapy, targeted therapy other than anti-HER-2 & endocrine therapy | 40 (3.9%) | 1 (3.0%) |
| Endocrine therapy & targeted therapy | 228 (9.1%) | 5 (0.4%) |
| Endocrine therapy & Anti-HER2 | 226 (99.1%) | 0 (0.0%) |
| Endocrine therapy & targeted therapy other than anti-HER-2 | 2 (0.9%) | 5 (0.4%) |
| Immunotherapy-based regimen | 0 (0.0%) | 0 (0.0%) |
| Other therapy | 0 (0.0%) | 0 (0.0%) |

Table S3: Number of metastatic lines of treatment according to mBC subtype

|  | **HR+ / HER2- (N=13283)** | **TN**  **(N=2845)** | **HR+ / HER2+ (N=2502)** | **HR - / HER2+ (N=1403)** | **Total**  **(N=20033)** |
| --- | --- | --- | --- | --- | --- |
| Median (IQR) | 3.0 (1.00, 4.000) | 2.0 (1.0, 3.0) | 2.0 (1.0, 4.0) | 2.0 (1.0, 3.0) | 2.0 (1.0, 4.0) |
| At least 1 line of treatment | 13283 (100.0%) | 2845 (100.0%) | 2502 (100.0%) | 1403 (100.0%) | 20033 (100.0%) |
| At least 2 lines of treatment | 9575 (72.1%) | 1861 (65.4%) | 1546 (61.8%) | 806 (57.4%) | 13788 (68.8%) |
| At least 3 lines of treatment | 6872 (51.7%) | 1141 (40.1%) | 1061 (42.4%) | 504 (35.9%) | 9578 (47.8%) |
| At least 4 lines of treatment | 4734 (35.6%) | 647 (22.7%) | 707 (28.3%) | 310 (22.1%) | 6398 (31.9%) |

Abbreviations: mBC, metastatic breast cancer; HR+, presence of hormone receptor; HR-, absence of hormone receptor); HER2+, human epidermal growth factor receptor 2 (HER2) protein overexpression; HER2-, no HER2 protein overexpression; TN,triple negative; IQR, interquartile range
